# Validation and Measurement Invariance of the Scale of Positive and Negative Experience (SPANE) in a Spanish General Sample

**DOI:** 10.3390/ijerph17228359

**Published:** 2020-11-12

**Authors:** Begoña Espejo, Irene Checa, Jaime Perales-Puchalt, Juan Francisco Lisón

**Affiliations:** 1 Department of Behavioral Sciences Methodology, University of Valencia, Av. Blasco Ibáñez, 21, 46010 Valencia, Spain; irene.checa@uv.es; 2 Alzheimer’s Disease Center, University of Kansas Medical Center, 3901 Rainbow Blvd., Kansas City, KS 66160, USA; jperales@kumc.edu; 3 Department of Medicine, Universidad Cardenal Herrera CEU, CEU Universities, 46010 Valencia, Spain; JUANFRAN@uchceu.es; 4 Centro de Investigación Biomédica en Red Fisiopatología Obesidad y Nutrición, Instituto Carlos III, Av. Monforte de Lemos, 3–5. Pabellón 11. Planta 0, 28029 Madrid, Spain

**Keywords:** confirmatory factor analysis, measurement invariance, psychometric properties, Spanish population, structural equation modeling, Scale of Positive and Negative Affects, well-being, health, quality of life, psychological assessment

## Abstract

Well-being has been measured based on different perspectives in positive psychology. However, it is necessary to measure affects and emotions correctly and to explore the independence of positive and negative affect. This cross-sectional study adapts and validates the Scale of Positive and Negative Experience (SPANE) with a non-probabilistic sample of 821 Spanish adults. A confirmatory factor analysis confirmed two related factors with two correlated errors. The average variance extracted was 0.502 for negative affect (SPANE-N) and 0.588 for positive affect (SPANE-P). The composite reliability was 0.791 for SPANE-N and 0.858 for SPANE-P. Measurement invariance analysis showed evidence of scalar invariance. Item-total corrected polyserial correlations showed values between 0.47 and 0.76. The path analysis used to test temporal stability, and the structural equation models used to test convergent and concurrent validity with other well-being measures, showed good fit. All path coefficients were statistically significant and over 0.480. For the validity models, the magnitude of the correlations was large and in the expected direction. The Spanish version of the SPANE show good psychometric properties. Future studies of emotional well-being in Spain can benefit from the use of this scale, and new studies must test cross-cultural invariance.

## 1. Introduction

Historically, the pursuit of happiness and human well-being have been central themes in literature, philosophy, and theology. Positive psychology has studied the psychological processes that underlie the search for happiness and that promote greater well-being [1,2,3]. Different constructs have been proposed to operationalize happiness and well-being, including satisfaction with life, positive and negative affect, and personal growth and development. These constructs are usually grouped into two different research perspectives: eudemonic and hedonic.

The eudemonic perspective, represented by psychological well-being (PWB), associates the well-being of the individual with the development of human potential. According to this perspective, well-being is intrinsically linked to the performance of activities congruent with the deep values of the individual that promote their personal development and growth [4] (Ryan and Deci, 2001). Happiness is conceived as a state of fullness and harmony in which well-being is understood as the perception of meaning and purpose in life, with personal growth and development being the main factors in the optimal functioning of the human being.

The hedonic perspective, characterized by subjective well-being (SWB), conceives the well-being of a person as the evaluation that people make about the satisfaction they experience in their lives and the balance between positive and negative affects [1,4]. To assess well-being from the hedonic perspective, the cognitive component must be taken into account, associated with the subjective–evaluative judgment that the person makes about their life, and the affective–emotional component [1]. In this sense, several instruments have been developed to assess well-being from the hedonic perspective: the Satisfaction with Life Scale [5], the Positive and Negative Affect Schedule [6], and the Scale of Positive and Negative Experience [7].

However, Aristotle already held that happiness should involve feeling emotions that people consider appropriate given their needs and motives. The Aristotelian model proposes that such people would be happier the more they feel the emotion they desire, even though that emotion is unpleasant. The traditional SWB model holds that people would be happier the less they feel that unpleasant emotion because pleasant emotions are considered as good and unpleasant emotions as bad.

The hedonic, eudemonic, and Aristotelian models of happiness probably overlap and complement each other to some extent. When individuals desire pleasant emotions, people are happier if they experience as much of a pleasant emotion as they desire. In this case, all the models match. However, when people desire unpleasant emotions, the models differ. Which emotions people consider desirable are different across situations, individuals, and cultures, as some authors have found [8]. Their research identified some cases in which people were happier even if they did not feel good. These findings show that happiness depends, in part, on the match between what people feel and what they want to feel. Therefore, people are happier when they experience emotions they desire, whether such emotions are pleasant or unpleasant. Individuals differ based on what they consider to be pleasant states [8]. Since value priorities differ across cultures [9], it is reasonable to assume that different emotions may be associated with happiness across cultures [10].

In order to put these models to the test and study which one of them best explains people’s well-being and happiness, it is necessary to correctly measure affects and emotions. However, ever since the beginning of the study of affect, there has been a continuous debate about the independence of positive and negative affect. Initially, researchers supported the idea that the positive and negative affect were independent [11]. However, later researchers defended that affect is a bipolar and unidimensional construct ranging from a maximum negative affect to a maximum [12,13]. According to the latter authors, positive and negative affect are not independent for four reasons: (1) the response scales used to measure affect as unipolar or bipolar constructs contain a significant amount of bias; (2) the correlation between positive and negative affect varies widely from −0.80 up to −0.92; (3) affect is a multidimensional construct encompassing not only a hedonic dimension but also dimensions such as drive and (4) the correlation between positive and negative affect increases when items ask about affect in the recent past [14,15]. According to Russell and Carrol, the illusion of the independence of positive and negative affect is a product of the compensation between intensity (positive correlation) and frequency (negative correlation). A comprehensive literature review of studies using the Positive and Negative Affect Schedule (PANAS) [6] found evidence both in favor and against the independence of affects [15].

The Scale of Positive and Negative Experience (SPANE) was created to offer a solution to the affect independence debate [7]. The SPANE is one of the most popular instruments used to measure affects worldwide. The original scale comprised 12 items, six positive (SPANE-P) and six negative experiences (SPANE-N). Both sets of items measure three general and three specific emotions encompassing a wide range of human experiences. Usually, existing surveys ask about specific feelings and weight them all identically, omitting important general feelings. Based on these scales, a person could feel generally positive or negative affect, but not feel many of the specific emotions listed on the scale. This person could score at an intermediate level on the scale despite feeling generally positive affect continuously. The SPANE, conversely, allows individuals to evaluate the degree to which these emotions are more desirable or pleasant for them. For instance, many of the items in the PANAS [6] are not considered emotions or feelings. The high arousal emotion items of the PANAS do not capture whether a person feels happy or contented. The SPANE, however, reflects all levels of arousal for both positive and negative feelings (e.g., happy or sad). The emotions used in the SPANE allow for capturing the major emotions included in most affect theories [7]. The authors of the SPANE incorporated two aspects to improve the assessment of affect: (1) framing response options in terms of the amount of time the person experiences each emotion, which is more related to well-being [16,17] and (2) limiting the time frame to the past four weeks, which improves recall of affect compared to other timeframes [18]. This scale is short enough to allow the respondent to recall actual experiences but avoids recalling only short-term emotions.

When the psychometric properties of the SPANE were studied, the authors conducted two principal axis factoring, one for the SPANE-N and one for the SPANE-P, among USA university students. Both factors showed a unidimensional structure with an explained variance of 53% and 61%, respectively. The positive and negative subscales showed a correlation of −0.60 with each other. The authors suggested a balanced score (SPANE-B) in which positive and negative items are subtracted and the score ranges from −24 to 24. In addition, the authors calculated normative scores, item-total correlations, and convergent and criterion validity with scales, including the Satisfaction With Life Scale (SWLS) [5], Revised Life Orientation Test (R-LOT) [19], and Positive And Negative Affect Scale (PANAS) [7], finding correlations between 0.30 and 0.70 in the expected direction. Studies have also shown that the SPANE has a higher predictive power of psychological well-being than the PANAS [20,21].

The SPANE has been validated in many countries, including Portugal [22], China [23], Japan [24], Canada [25], Turkey [26], India [27], Italy [28], South Africa [29], Germany [30], Peru [31], Chile [32], Greece [33], Serbia [20,21], Romania [34], and Mexico [35], using non-probabilistic samples of students (undergraduate or college), adolescents, young adults, or workers, ranging from 401 college students [26] (Telef, 2015) to 21,322 workers [23] (Li et al., 2013). All studies (except South African) conducted a confirmatory factor analysis (CFA), finding a two-factor solution as the best one. However, the Chinese study found a two-factor solution with correlated errors to obtain the best fit. Regarding measurement invariance, the Indian version showed configural invariance between adolescents and adults. The Chinese study showed strict invariance across gender, age, and marital status, and strong measurement invariance across education level and income level. The Italian version obtained strict measurement invariance across administration methods (paper and pencil and Internet) and strong measurement invariance across different groups (unemployed individuals seeking work and a healthy control group). The Greek and Romanian versions showed strict measurement invariance by gender, in line with the Chinese one. Only in the Serbian version did some items of both subscales not show measurement invariance across gender [20,21]. Reliability of both subscales was satisfactory in all the studies, with Chronbach’s alpha coefficients ranging from 0.71 for the SPANE-N to 0.92 for the SPANE-P. The less commonly studied test–retest reliability has shown good stability. Validity has been tested by calculating correlations among different subjective well-being scales and the two factors of the SPANE scale. These correlations have mostly been statistically significant and in the expected direction.

The SPANE has shown promising psychometric properties (reliability, validity, and gender measurement invariance) in different populations and has been used widely in different well-being studies across the globe. The SPANE has the potential to allow researchers to test the different models of people’s well-being and happiness. Therefore, this paper aims to adapt the SPANE to the Spanish population and study its psychometric properties with a large Spanish sample. Furthermore, we study the SPANE’s measurement invariance for gender and use structural equation modeling (SEM) to study its factor structure, the test–retest reliability, and correlations with other well-being measures. We use the SEM methodology to study validity because it allows us to estimate the error contained in the observed scores, as is done when estimating the confirmatory factor analysis (CFA). If the correlation between the total scores of the different measures used to study validity is calculated, correlations between observed scores will be obtained. However, since these observed scores contain errors, these correlations will underestimate the validity. If the correlation between the latent variables is calculated using an SEM, the error (which is estimated separately) will be extracted from these correlations, as occurs when estimating a CFA. For this reason, performing an SEM is a more reliable way to estimate the relationship between measures. On the other hand, conducting the exploratory factor analysis is not necessary for the present paper, for at least two reasons. First, there is a strong theoretical expectation for the two-factor structure of the SPANE. Second, the two-factor structure has been supported in all previous studies.

## 2. Materials and Methods

### 2.1. Participants and Procedure

The Spanish adaptation process of the SPANE was conducted using the International Test Commission (ITC) criteria [36,37,38]. These authors stress the need to ensure conceptual, linguistic, and metric equivalence. The adaptation of the adjectives of the scale was conducted using the translation–back translation method by two bilingual translators. Discrepancies between both translators were reported to the first author of the scale, Dr. Edward F. Diener, for clarification. The final version of the adjectives of the scale in Spanish is shown in the Appendix A. Participants were recruited via email including an explanation of the study and a link to LimeSurvey, an open-source survey tool (www.limesurvey.org). Participants had to read and accept an online informed consent before entering the study. They then responded to the socio-demographic variables and to all the questionnaires. A retest was conducted one month later to study test–retest reliability, by sending again the link to the survey to all the participants in the test stage. The study was conducted in compliance with Spanish legislation (Ley Orgánica 3/2018, 5 December) and the code of ethics for research involving human subjects, as outlined by the Universitat de València Human Research Ethics Committee (ACGUV194/2006).

Participants were included if they identified themselves as Spaniards in the survey and if they were at least 18 years of age. The final sample comprised 821 participants. The average age was 30 years (SD = 12.1, median = 25, mode = 21, quartile 25 = 21, quartile 75 = 36) ranging from 18 to 71 years of age. Of the sample, 32.5% were men, 49.1% had completed at least college/university education, 46.5% had completed high-school level education, and 4.4% had finished compulsory secondary studies or had only primary studies. Regarding marital status, 57.7% were single, 38.7% were married or cohabiting, and 3.6% were divorced or widowed. Regarding the working status, 37.3% were students only, 22.3% were students and had sporadic or part-time jobs, 33% were employed or self-employed, 4.8% were unemployed, 1.2% were inactive, and 1.5% were retired. The retest sample was composed of 52 participants. Of them, 32.7% were men. The average age was 35.06 years (SD = 12) ranging from 21 to 66 years of age. No more socio-demographic data were collected for the retest sample.

### 2.2. Instruments

Scale of Positive and Negative Experience (SPANE). This is a twelve-item instrument describing a wide range of human experiences [7]. The scale comprises six positive (SPANE-P) and six negative experiences (SPANE-N) measuring three general and three specific emotions. The instrument uses a five-point frequency rating scale ranging from 1 (very rarely or never) to 5 (very often or always). Total scores range from 6 to 30 with high scores indicating high positive or high negative affect. SPANE-P and SPANE-N can be subtracted to obtain a balance measure that ranges from −24 to 24.

Flourishing Scale (FS). This is an eight-item instrument describing important aspects of human functioning including positive relationships, feelings of competence, and having meaning and purpose in life [7]. The instrument uses a seven-point Likert scale ranging from 1 (strongly disagree) to 7 (strongly agree). Total scores range from 8 to 56 with high scores indicating respondents viewing themselves in positive terms in important areas of functioning. This instrument has been validated in a general sample of Spanish adults and has shown an internal consistency of 0.85 [39]. The internal consistency in this sample is 0.86.

Positive And Negative Affect Schedule (PANAS). This scale measures two domains, positive and negative affect [6]. This 20-item instrument uses a five-point Likert scale ranging from 1 (not at all) to 5 (extremely). Items include “interested”, “excited”, “ashamed”, or “irritable”. Scores range from 10 to 50, higher scores indicating higher affect. The Spanish version used in the present study has been validated in a large sample of Spanish adolescents and young adults showing internal consistency scores from 0.80 to 0.86 [40]. In our total sample, internal consistency was 0.848 for positive affect and 0.860 for negative affect.

Satisfaction With Life Scale (SWLS). This is a five-item instrument designed to measure global cognitive judgment of satisfaction with one’s life [5]. Participants indicate how much they agree or disagree with each of the five items using a seven-point scale that ranges from 7 (strongly agree) to 1 (strongly disagree). Scores range from 7 to 35, higher scores indicating higher satisfaction. This instrument has been validated in a general sample of Spanish adults and has shown an internal consistency of 0.858 [41]. Internal consistency in the present sample is 0.86.

Life Orientation Test-Revised (LOT-R). This questionnaire has been used to measure optimism and pessimism [19]. The scale comprises ten items, four control items, three pessimism items, and three optimism items. Each item of the LOT-R is answered on a five-point Likert scale that ranges from 1 (strong disagreement) to 5 (strong agreement). The scores range from 0 to 12, higher scores in both subscales indicate high optimism or high pessimism, respectively. This scale has been validated in a general sample of Spanish adults and has shown good psychometric properties [42]. Internal consistency in this sample is 0.772 for the optimism subscale, and 0.715 for the pessimism subscale.

### 2.3. Data Analysis

To study the SPANE’s factor structure, we estimated three CFAs to test a one-factor model, a two independent factors model, and a two correlated factors model. Due to the sensitivity to the sample size of the χ^2^ goodness of fit test, we used different indices to determine model fit: The comparative fit index (CFI), the root mean square error of approximation (RMSEA), and the standardized root mean squared residual (SRMR). CFI values of 0.90 and RMSEA values above 0.06 and below 0.08 are indicative of acceptable model fit. CFI values of 0.95 or above and RMSEA values below 0.05 are indicative of good empirical fit, and SRMR values close to 0.08 are indicative of good model fit [43,44,45]. We calculated reliability for both subscales using the composite reliability index and the average variance extracted index [46].

We conducted measurement invariance for gender by assessing three nested models with increasing restrictions using the second sample. The first model assessed configural invariance, which allowed for the free estimation of all parameters for each group. The second model assessed metric invariance, nested within the configural invariance model, which added the restriction of invariant factor loadings between groups. The third model assessed scalar invariance, nested within the metric invariance model, which added the restriction of the intercepts of invariant items between groups. Increases in CFI and RMSEA values greater than 0.010 indicated a lack of invariance [47]. To study the convergent validity, we specified a structural equation model (SEM) including the items and their underlying factors of the SPANE, the FS, and the PANAS. We tested concurrent validity with another structural equation model, including the items and their underlying factors of the LOT (optimism and pessimism subscales) and the items of the SWLS.

We used Mplus 8.1 (Muthén & Muthén, Los Angeles, CA, USA) to carry out these analyses [48], considering all observed variables as ordinal and estimating parameters using polychoric correlations [49] and maximum likelihood robust (MLR) estimation. Even though MLR is not designed specifically for ordinal data, some authors have suggested the use of MLR estimation in CFA or CFA-based models with a non-normal distribution of data, and if the number of response categories for each item is sufficiently large (equal to or greater than five) [50,51]. In this case, it can be assumed that observed data are approximately continuous, and the variability in parameter estimates is small [51,52]. MLR has generally less biased standard error estimates and good recovery of the population inter-factor correlations [53].

Instead of Pearson correlations, we calculated corrected item-total polyserial correlations for the items in each subscale [54], as indicators of corrected homogeneity indices for items with ordinal response scales [49,50]. We specified a path analysis to study test–retest reliability in which we used total scores of SPANE-P and SPANE-N at Time 1 as predictors, and total scores of SPANE-P and SPANE-N at Time 2 as criteria. We considered correlations between SPANE-P and SPANE-N at Time 1, and between SPANE-P and SPANE-N at Time 2 for the model. We used the maximum likelihood (ML) estimation method to estimate parameters. We also used χ^2^ statistic SRMR to determine model fit, with a cutoff value close to 0.08 as indicative of good empirical fit [44]. We used IBM SPSS 26 (IBM Corporation, New York, NY, USA) to describe sociodemographic variables [55].

## 3. Results

### 3.1. Dimensionality

Table 1 shows the goodness of fit indicators of the models tested. Two-dimensional models showed a better fit compared to the one-dimensional model. However, model 3 showed only an acceptable fit. For this model, two modification indices (MI) showed high values, between items 1 and 2 (MI = 68.920), and between items 3 and 4 (MI = 77.647). These items correspond to the adjectives 1 (negative) and 2 (positive), and items 3 (bad) and 4 (good), with opposite meanings. When a person considers that they are “often” very positive, they are expected to respond that they are “almost never” negative. The same applies to good and bad. Model 4 allowed for the correlation of residuals between these pairs of items. Models 3 and 4 (correlated factors) had a better fit than model 2 (independent affect factors), but model 4 showed the best goodness of fit: CFI (0.950), RMSEA (0.061), RMSEA 90% CI [0.052, 0.070], and SRMR (0.043), even though χ^2^/df was over 2. The average variance extracted index was acceptable for negative affect (0.502) and good for positive affect (0.588), above the minimum level of 0.50. The composite reliability index was good for negative affect (0.791) and for positive affect (0.858).

**Figure 1:**
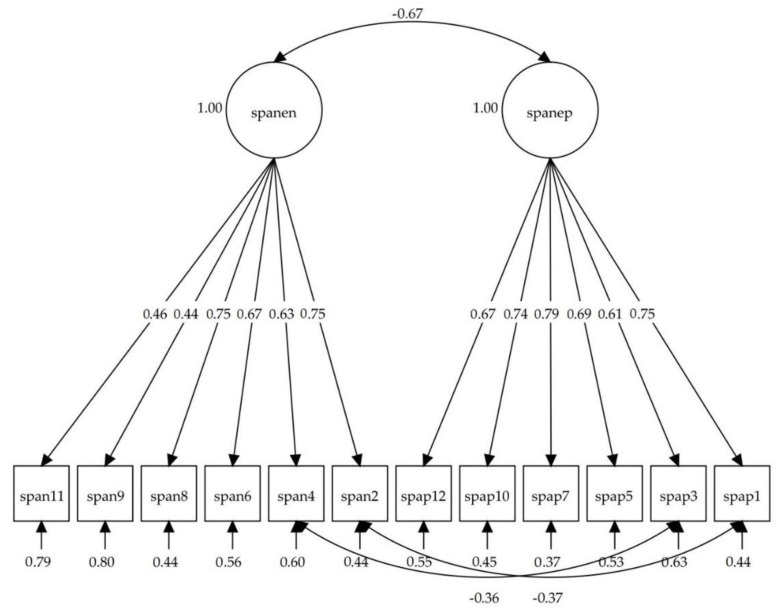
Path diagram with a summary of the fourth model, obtained from the items of the Scale of Positive and Negative Experience (SPANE); neg: SPANE-N; pos: SPANE-P.

Figure 1 shows the path diagram with standardized factor loadings of the fourth model for the Spanish version of the SPANE, as well as the standardized correlation among latent variables. All factorial loadings were statistically significant (*p* < 0.001), and ranged from 0.444 to 0.751 for the SPANE-N, and from 0.606 to 0.792 for the SPANE-P. The correlation between the positive and negative factors was −0.673, high but significantly different from one (1 > 2SE), which indicates they are empirically discriminable factors. Residuals remained close to zero.

### 3.2. Measurement Invariance for Gender

Table 2 shows the results for the measurement invariance models by gender. The results show that the SPANE had metric invariance by gender, and the fit of model 4 (with correlated errors for items 1 and 2, and for items 3 and 4) for men and women was good. As can be seen, ΔCFI and ΔRMSEA values are lower than 0.010 for the three models. The latent mean values were fixed to zero for men and women had more negative affect than men (*b* = 0.198, *z* = 3.610, *p* < 0.01) and showed no differences in positive affect (*b* = −0.014, *z* = −0.292, *p* = 0.771).

### 3.3. Test–Retest Reliability and Item-Total Corrected Polyserial Correlations

**Figure 2:**
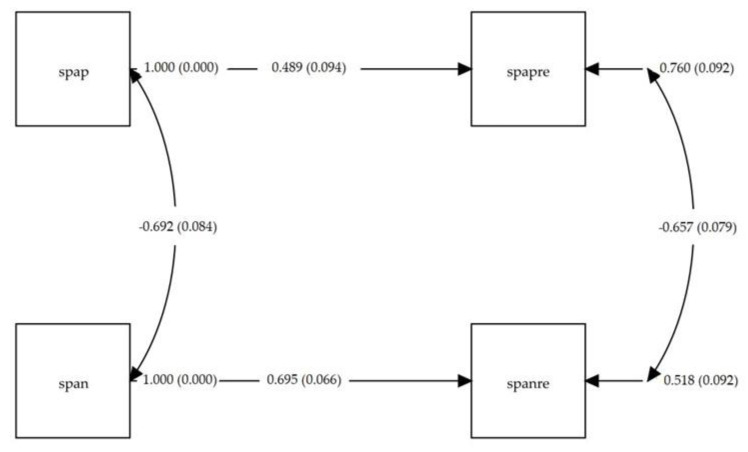
Path diagram of the test–retest model for the Scale of Positive and Negative Experience (SPANE) total scores, with correlations and regression coefficients (standard error); spap: test total score for the SPANE-P; span: test total score for the SPANE-N; spapre: retest total score for the SPANE-P; spanre: retest total score for the SPANE-N. All path coefficients were statistically significant (*p* < 0.001).

Figure 2 shows the test–retest path analysis. The model showed very good fit: χ^2^(2) = 0.038 (*p* = 0.826), and SRMR = 0.037 (notably below 0.08). All path coefficients were statistically significant (*p* < 0.001). Item-total corrected polyserial correlations showed good values, that ranged from 0.643 to 0.754 for the SPANE-P, and from 0.470 to 0.703 for the SPANE-N.

### 3.4. Convergent and Concurrent Validity

**Figure 3:**
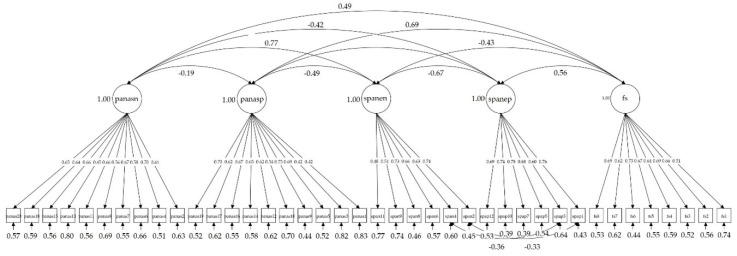
Path diagram for the convergent validity model with correlation coefficients among latent variables and factorial loadings; panasn: Positive and Negative Affects Schedule (negative); panasp: Positive and Negative Affects Schedule (positive); spanen: Scale of Positive and Negative Affects (negative); spanep: Scale of Positive and Negative Affects (positive); fs: Flourishing Scale.

Regarding the validity models, the two-factor model for the SPANE with correlated errors for items 1 and 2, and for items 3 and 4, was fitted to the data, as this was the model that showed the best fit when studying the factor structure. The convergent validity model with the FS and the PANAS subscales showed acceptable fit to data: χ^2^(728) = 3078.754 (*p* < 0.001), χ^2^/gl = 4.23, CFI = 0.804, RMSEA = 0.063, RMSEA 90% CI [0.061, 0.065], and SRMR = 0.072. However, three modification indices showed high values, between item 7 (scared) and 20 (afraid) of the PANAS (MI = 224.098), between item 9 (afraid) of the SPANE and 7 (scared) of the PANAS (MI = 209.200), and between item 9 (afraid) of the SPANE and 20 (afraid) of the PANAS (MI = 135.244). For these pairs of items, the meaning is practically the same, and probably that is the reason for such big modification indices. Therefore, we fitted the model again to the data estimating the correlation of residuals between these pairs of items. This model showed a better fit: χ^2^(725) = 2526.180 (*p* < 0.001), χ^2^/gl = 3.48, CFI (0.850), RMSEA (0.055), RMSEA 90% CI [0.053, 0.058], and SRMR (0.070). Figure 3 shows the path diagram for the convergent validity model. All factorial loadings were statistically significant (*p* < 0.001), and ranged between 0.65 and 0.70 for the Positive and Negative Affects Schedule (negative), between 0.42 and 0.75 for the Positive and Negative Affects Schedule (positive), between 0.48 and 0.74 for the Scale of Positive and Negative Affects (negative), between 0.68 and 0.78 for the Scale of Positive and Negative Affects (positive) and between 0.51 and 0.75 for the Flourishing Scale.

**Figure 4:**
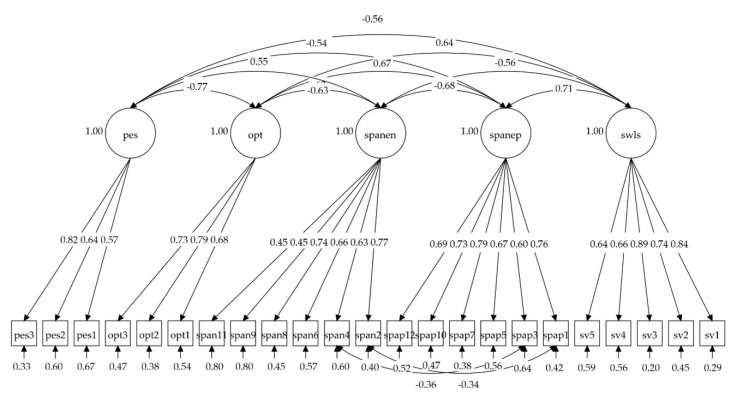
Path diagram for the concurrent validity model with correlation coefficients among latent variables and factorial loadings; pes: Life Orientation Test (pessimism subscale); opt: Life Orientation Test (optimism subscale); spanen: Scale of Positive and Negative Affects (negative subscale); spanep: Scale of Positive and Negative Affects (positive subscale); swls: Satisfaction with Life Scale.

The concurrent validity model with the optimism and pessimism subscales of the LOT-R, and with the SWLS, showed very good fit to data: χ^2^(218) = 558.541 (*p* < 0.001), CFI = 0.951, RMSEA = 0.044, RMSEA 90% CI [0.040, 0.049], and SRMR = 0.042. Figure 4 shows the path diagram for the concurrent validity model. All factorial loadings were statistically significant (*p* < 0.001). Table 3 shows the correlations among latent variables for both models. The magnitude of the correlations can be considered large and in the expected sense. All of them were statistically significant (*p* < 0.001) and ranged between 0.57 and 0.82 for the Pessimism Subscale, between 0.68 and 0.73 for the Optimism Subscale, between 0.45 and 0.77 for the Scale of Positive and Negative Affects (negative), between 0.60 and 0.79 for the Scale of Positive and Negative Affects (positive), and between 0.64 and 0.89 for the Satisfaction with Life Scale.

## 4. Discussion

Well-being has been measured based on different perspectives in positive psychology. However, it is necessary to correctly measure affects and emotions and to explore the independence of positive and negative affect. One of the most popular instruments used to measure affects is the Scale of Positive and Negative Experience (SPANE) [7]. In this study, we have adapted and validated the Scale of Positive and Negative Experience (SPANE) in Spanish adults. We have used structural equation modeling to test the psychometric properties of this scale. Structural equation modeling is a more powerful approach than classical test theory, offering a more reliable and valid estimate of the psychometric properties of a test.

Findings from this study show that the version of the SPANE among Spaniards has good construct, convergent, and concurrent validity, as well as good temporal stability, reliability (Composite Reliability Index and Average Variance Extracted Index) and scalar invariance for gender. Specifically, we found that the best model for the SPANE for Spaniards is the one that proposes a structure of two related factors, with positive and negative affects highly correlated, but different from each other. This high correlation between the two types of affect has also been found in the original version [7] and in the Chinese, Italian, Portuguese, Serbian, and Mexican adaptations [20,21,22,23,28,35], despite the lower association found in the Japanese version [24]. However, as our study and others suggest, the fact that there is a high correlation between both types of affect does not imply that affect should be measured one-dimensionally.

In our study, we found that the model with two correlated factors with correlated errors had the best fit, which is in line with the Chinese validation of the SPANE [23]. Positive and negative feelings seem to be statistically separable into two strongly inversely correlated factors even when measurement error is controlled for. Allowing for the correlation between both factors in the case of SPANE and PANAS seems correct given that they provide a theoretical explanation about situation—general or specific—affect, and the item factor weights do not change substantially from one model to another and only 15% of the possible total error terms are controlled.

The authors confirm with this study the SPANE’s scalar invariance for gender in a Spanish population. Validation studies from Greece [33], China [23], and Romania [34] showed strict invariance. However, the Serbian study [21] did not show invariance by gender for all the items. Strong invariance allows for comparing the latent means. In the present study, we have found differences in negative affect, being greater for women, which is in line with similar studies [21,32].

Both affects measure three general and three specific emotions encompassing a wide range of human experiences. Usually, existing surveys ask about specific feelings and weight them all identically, omitting important general feelings. Based on these scales, a person could feel generally positive or negative affect, but not feel many of the specific emotions listed on the scale. This person could score at an intermediate level on the scale despite feeling generally positive affect continuously. The SPANE, conversely, allows individuals to evaluate the degree to which these emotions are more desirable or pleasant for them. For instance, many of the items in the PANAS [6] are not considered emotions or feelings. The high arousal emotion items of the PANAS do not capture whether a person feels happy or contented.

The SPANE reflects all levels of arousal for both positive and negative feelings, and the emotions used in this scale allow for capturing the major emotions included in most affect theories [7]. Moreover, the SPANE adds some improvements to the assessment of affect. On one hand, it uses response options in terms of the amount of time the person experiences each emotion (always, never, etc.), focusing on frequency rather than on the intensity of affect [56], which reduces cognitive bias [7,18] and is more strongly related to well-being measures such as life satisfaction than the intensity of those feelings [17]. On the other hand, the timeframe is limited to the past four weeks, which is short enough to allow the respondent to recall actual experiences rather than rely on general self-concept [7].

Other interesting approaches have emerged to decrease cognitive bias when measuring affects, such as the Ecological Momentary Assessment (EMA) and the Day Reconstruction Method (DRM) [57]. The EMA involves repeated sampling of experiences and mood in real time in the natural environment. However, the respondent burden with this method is high, and assessments may be biased (increases fatigue and decreases motivation). Although ecological validity is maximized, there are some circumstances in which collection of EMA data is difficult, as with some high-stress occupational groups. The DRM studies the retrospective recall of the established period as a sequence of episodes. Each episode has its onset and duration, location, social interaction, and activity. Participants also rate the episodes on a series of affect scales. Even though the DRM involves recall, it is designed to increase the accuracy of emotional recollection by involving retrieval of the specifics of each episode [58] (Robinson and Clore 2002). Both methods seem promising in reducing this type of information bias, however, in both methods, the respondent burden can be high, whereas the SPANE only takes a few minutes to complete and uses a short time-period of recall. Both approaches can probably be complementary with the use of the SPANE.

There are more valuable implications for this study regarding the use of this scale. Spain and other Spanish-speaking countries can benefit from the Spanish validation of the SPANE to assess the efficacy of interventions aiming to improve well-being and use it to measure the positive affects. The SPANE can be used in randomized trials to evaluate the efficacy of interventions on health. For instance, some trials have shown that a six-week yoga intervention [59] can improve positive affect (SPANE scores) in different populations and settings. SPANE can be used, too, to assess convergent validity in studies that develop more culture-specific instruments, like in a recent Indian study that developed a scale to understand human functioning from a purely Indian perspective called the Ashtanga Yoga Hindi Scale [60], and the SPANE was used to test the validity of this new specific scale. Finally, institutions such as the European Union have shown an increasing interest in the measurement and improvement of the well-being of their population in the past decade as part of an active aging strategy [61]. Since the SPANE has been validated in multiple languages and countries, is brief, and is psychometrically well developed, it has the potential to be implemented in wide international population health surveys to compare levels of affect and to assess risk and protective factors.

Limitations of this study include not measuring discriminant validity, which leaves the question of which constructs are unrelated to the SPANE unanswered. Since the sample is not representative of the population, this can lead to problems with generalization of the results. To our knowledge, no version of the SPANE has been validated using probability samples. Future validations should address this issue by using a probabilistic sample. It would be desirable, too, to develop norms for different groups in large and probability samples. On the other hand, responsiveness to change was not assessed in this study, and future studies should assess whether the SPANE is associated with health outcomes. Similarly to chronic stress, the frequency of positive and negative stress could be associated with a psycho-neuro-immunological system that is related to disease [62]. For this reason, these studies would benefit from the assessment of biomarkers including cortisol and functional magnetic resonance. Finally, despite the benefits of the timeframe used in the SPANE questions, comparisons between the current version of the SPANE and a version using the ecological momentary method should be conducted to quantify potential recall bias.

## 5. Conclusions

The present study provides a version of a widely used measure of perceived well-being, the SPANE, for Spaniards. The study details the back translation process and study measurement invariance by gender, as well as factor structure and psychometric properties using structural equation modeling methodology. Our results indicate that the Spanish adaptation of the SPANE has adequate psychometric properties, similar to those in previous studies. The Spanish population and researchers can benefit from this short and easy-to-understand scale that can be implemented in a variety of studies including randomized trials, population surveys, or the design and validation of related instruments. This scale can be an efficient and valuable tool for clinicians and researchers in Spain.

## Figures and Tables

**Table 1:** Goodness of fit indicators of the models of affect tested using confirmatory factor analysis.

	χ^2^	df	χ^2^/df	CFI	RMSEA	RMSEA 90% CI	SRMR
(1) 1 factor	695.205 *	54	3.39	0.793	0.121	0.113, 0.129	0.076
(2) 2 factors (P and N)	648.994 *	54	12.02	0.808	0.117	0.109, 0.125	0.198
(3) 2 correlated factors (P and N)	354.873 *	53	6.53	0.902	0.084	0.076, 0.092	0.048
(4) 2 correlated factors (P and N) with correlated errors ^a^	204.428 *	51	4.01	0.950	0.061	0.052, 0.070	0.043

*Note*. df = degrees of freedom; CFI = comparative fit index; RMSEA = root mean square error of approximation; SRMR = standardized root mean squared residual; P = Scale of Positive and Negative Experience (Positive); N = Scale of Positive and Negative Experience (Negative). ^a^ Model with correlated errors for items 1 (negative) and 2 (positive), and items 3 (bad) and 4 (good) * *p* < 0.001.

**Table 2:** Measurement invariance models of the SPANE by gender (reference group: men).

Model	χ^2^	df	Δχ^2^	Δgl	CFI	RMSEA	RMSEA 90% CI	ΔCFI	ΔRMSEA
Men	114.709 *	51			0.950	0.064	0.048, 0.080		
Women	157.537 *	51			0.958	0.056	0.046, 0.065		
Configural	271.327 *	102	-	-	0.955	0.058	0.050, 0.067	-	-
Metric	274.807 *	112	3.246	10	0.957	0.054	0.046, 0.063	0.002	−0.004
Scalar	319.048 *	122	46.192	10	0.948	0.057	0.050, 0.065	0.009	0.003

df = degrees of freedom; Δχ^2^ = chi square increase; Δgl = increase in degrees of freedom; CFI = comparative fit index; RMSEA = root mean square error of approximation; ΔCFI = CFI increase; ΔRMSEA = RMSEA increase. * *p* < 0.001.

**Table 3:** Correlation coefficients (standard errors) between SPANE subscales and well-being measures in the convergent and the concurrent validity models.

	Convergent Validity Model				Concurrent Validity Model			
	SPANE-P	SPANE-N	PANAS-P	PANAS-N	SPANE-P	SPANE-N	SWLS	OPT
FS	0.560 (0.057)	−0.421 (0.053)	0.492 (0.058)	−0.282 (0.053)				
PANAS-N	−0.433 (0.043)	0.763 (0.039)	−0.187 (0.050)					
PANAS-P	0.684 (0.030)	−0.481 (0.041)						
SPANE-N	−0.673 (0.035)							
SPANE-N					−0.676 (0.035)			
SWLS					0.711 (0.027)	−0.559 (0.037)		
OPT					0.673 (0.029)	−0.632 (0.031)	0.638 (0.032)	
PES					−0.535 (0.039)	0.550 (0.039)	−0.560 (0.039)	−0.766 (0.032)

*Note*. FS = Flourishing Scale; PANAS-N = Positive and Negative Affects Scale (negative subscale); PANAS-P = Positive and Negative Affects Scale (positive subscale); SPANE-P = Scale of Positive and Negative Experience (positive subscale); SPANE-N = Scale of Positive and Negative Experience (negative subscale); SWLS = Satisfaction with Life Scale; OPT = Life Orientation Test (optimism subscale); PES = Life Orientation Test (pessimism subscale). All correlations were statistically significant (*p* < 0.001).

## Appendix A

Spanish version of the Scale of Positive and Negative Affects (SPANE).

Por favor, piense en lo que ha experimentado durante las últimas cuatro semanas. Luego, usando la escala que se le proporciona, describa en qué medida experimentó cada una de las siguientes emociones. Para cada enunciado, seleccione un número del 1 al 5.

Muy raramente o nunca

Casi nunca

A veces

A menudo

Muy a menudo o siempre

Positivo

Negativo

Bueno

Malo

Agradable

Desagradable

Feliz

Triste

Asustado

Alegre

Enfadado

Satisfecho
